# Geographical Variation in Body Size in the Asian Common Toad (*Duttaphrynus melanostictus*)

**DOI:** 10.3390/life13112219

**Published:** 2023-11-17

**Authors:** Kunhao Zhang, Duojing Qiu, Li Zhao, Chengzhi Yan, Long Jin, Wenbo Liao

**Affiliations:** 1 Key Laboratory of Southwest China Wildlife Resources Conservation (Ministry of Education), China West Normal University, Nanchong 637009, China; 18482369270@163.com (K.Z.); qiuduojing@126.com (D.Q.); lizhao_688@163.com (L.Z.); ychzhi@aliyun.com (C.Y.); 2 Key Laboratory of Artificial Propagation and Utilization in Anurans of Nanchong City, China West Normal University, Nanchong 637009, China

**Keywords:** Asian common toad, body size, age structure, geographic variation

## Abstract

The geographic variation in life-history traits of organisms and the mechanisms underlying adaptation are interesting ideas in evolutionary biology. This study investigated age and body size of the Asian common toad (*Duttaphrynus melanostictus*) among five populations along a geographical gradient. We found that geographical variation in age was non-significant among populations but there was a significant and positive correlation between mean age and body size. Although the body size values at 1043 m are quite different from other sites, after controlling for age effects, there was a significant positive correlation between altitude and body size. Our findings followed the predictions of Bergmann’s rule, suggesting that the body size of *D. melanostictus* is potentially influenced by the low air temperatures at higher altitudes.

## 1. Introduction

The environmental factors temperature and rainfall are two important selective forces that lead to geographic variation in morphological, physiological and life-history characteristics [1,2,3,4,5,6,7,8,9,10,11,12,13,14,15,16,17,18,19]. Especially, temperature is a key factor that leads to geographic variation in age and body size in animals because the responses to environmental temperature along geographic gradients are likely influenced not only by energy storage but also by food intake [13,20,21]. In addition, body size variation across populations is regarded as an outcome of sexual and natural selection [22,23,24]. By contrast, larger body size in females arises as a result of the strong fecundity selection where increased reproductive investment favors large females [25,26]. 

Body size is an important life-history trait that affects the success of ecological interactions and, ultimately, reproductive fitness [27,28]. Body size variation results from changing growth rates, shifting age structure or a combination of the two, which affects population-, community- and ecosystem-level dynamics [29,30]. There is evidence that a small body disadvantages males attempting to obtain a mate during the competitive interactions within a species [31,32]. In the meantime, a substantial reduction in body size results in populations that exhibit heightened vulnerability to collapse, as diminished body size significantly declines both fecundity and survival within the natural environment [33]. 

Because there are significant changes in body size across temporal and spatial scales, extensive studies have been conducted to identify factors contributing to body size variation, with a particular focus on environmental temperature [18,19,20,27]. Indeed, temperature affects metabolism, which has an effect on body size through its influence on energy allocation to growth, activity and reproduction in organisms [34]. In terms of geographic variation in body size, Bergmann’s rule states that a larger size is often obtained in colder temperatures than in warmer temperatures within or among species. Bergmann’s rule has been largely supported in homeothermic species. These patterns hold true in endotherms based on a positive correlation between basal metabolic rate and body mass [35]. In particular, the explanation for Bergmann’s rule is that larger-bodied animals possess a smaller surface-to-volume ratio, where heat conservation is more effective [36]. However, larger individuals tend to lose more energy to the environment than smaller individuals which obey the generality of Bergmann’s rule [37]. Numerous researchers have used different animal groups to test this hypothesis, and some results showed that some endothermic animals follow Bergmann’s rule [38,39,40,41,42,43] while others follow the inverse of Bergmann’s rule [44,45,46].

Anurans, as an ideal model, were used for studying geographic variation in body size because body temperature depends tightly on environmental temperature [29]. There is evidence for intra-specific studies on the effect of temperature on body size in frogs [27,46]. However, there is much controversy on how environmental temperature influences variation in body size in frogs. For instance, species living at lower temperatures in high altitude and/or latitude areas have larger body sizes compared to those living at high temperatures in low altitude and/or latitude areas, following Bergmann’s rule [27,47,48], while other species contrast this [13,49]. Moreover, age, as one of the basic life-history traits, affects body size variation in an organism. Usually, organisms under harsh environments will allocate more energy and time into growth, so that individuals become older and have larger bodies [39,50,51]. Indeed, most species display positive correlations between age and body size [13,42].

The Asian common toad (*D. melanostictus* ) is one of the most prevalent anurans in Asia [52]. This species grows and breeds in various habitats, including marshes, puddles, man-made ponds and temporary ponds, providing it an ideal model for studying body size variation. Its most distinctive feature lie in the black bony spines protruding from its snout. With strong adaptability, this species can thrive across a range of altitudes from 10 to 2000 m and breeds between March and August [53]. Here, we investigated patterns of variation in age and body size in *D. melanostictus* among five populations along a geographical gradient. Altitude or latitude have different connotations, where altitude has elevation shifts leading to not only temperature shifts but also exposure to sun and oxygen, and latitude shifts may be more related to length of day and temperature [54,55,56,57]. We first examined the difference in age across altitudes or latitudes. We then explored the relationships between body size and altitude or latitude among populations when controlling the age effect to verify whether Bergmann’s rule applies to this species, and we predicted an increase in body size with increasing altitude or latitude in the species.

## 2. Materials and Methods

### 2.1. Study Species

The Asian common toad is a medium-sized anuran, with the female having a larger body size than males. The toad is widely distributed in southern China where they live in holes in the ground during the day and forage for insects at night [58]. It is a lekking species, where males actively wait for females for mating in pools during the night in breeding season. Upon the females’ arrival at the breeding pools, males can promptly approach and engage in clasping behavior [52]. The females usually lay a larger number of eggs, ranging from 2500 to 4000 eggs in the spawning site. There are some poison glands on the dorsum skin in this species which can be used to avoid predators such as snakes [53].

### 2.2. Sample Site

Fieldwork was conducted from April to August between 2018 and 2020. We studied five *D. melanostictus* populations including Midu, Mouding and Pingbian from the Yunnan province, Yuanling from the Hunan province and Pingjiang from the Guizhou province. These study sites significantly display variations in altitude, mean temperature and mean precipitation [52]. The three sites in Yunan included Midu at an altitude of 1673 m, Mouding at an altitude of 1771 m and Pingbian at an altitude of 1043 m [52]. The vegetations at these three sites were characterized by *Taxus chinensis*, *Anoectochilus pingbianensis*, *Rhododendron platyphyllum*, *Cheirostylis pingbianensis*, *Rungia pinpienensis*, and *Ophiorrhiza pingbienensis*. The Pingjiang population was found along some paddy fields at an altitude of 275 m. The sampling area was about 100 m long and 60 m wide. The vegetation at this site was characterized by *Begonia rongjiangensis* and *Alsophila spinulosa*. For the Yuanling population, the paddy fields were located an altitude of 275 m and we found *Cephalotaxus oliveri*, *T. chinensis* and *Manglietia patungensis* in the area.

### 2.3. Sample Collection

We randomly captured 116 males and 37 females along sampling lines (length 500 m; width 10 m) at night using flashlight illumination during the breeding season from five populations (Table 1). The toad museum number information at different collecting sites is shown in Supplementary Materials Table S1. We diagnosed the toad as *D. melanostictus* on the basis of their morphological characteristics and body color [53]. We identified their sex by their secondary sexual characteristics (e.g., eggs in females and nuptial pads in males [52]). We euthanized all specimens using single-pithing, and then preserved them in 4% phosphate-buffered formalin for tissue fixation. The toes were clipped after the specimens were euthanized. All individuals with a specific museum number were stored in a public natural history collection in China West Normal University. The specimens used in this study were collected with permission from the China West Normal University Ethical Committee for Animal Experiments (CWNU-20001).

### 2.4. Body Size Measurement

The body mass of each individual was measured using an electronic balance with an accuracy of 0.01 g. Then, we used the vernier caliper to measure snout-vent length (SVL) of each individual with an accuracy of 0.01 mm [58,59]. The measurements and records of each toad were measured twice by two persons for minimizing error. We clipped the longest phalanges of all individuals from the right hindlimb and then stored them in 4% neutral buffered formalin in order to determine age structure using skeletochronology [36].

### 2.5. Age Determination

The most commonly used method for determining the age of amphibians is skeletochronology [36,59,60,61,62,63,64]. We used the paraffin section method and Harris’s haematoxylin stain to obtain histological sections of the phalanges [65]. We first removed the skins and muscles of each digit, and washed the remaining bones in water for two hours. We then decalcified the bones in 5% nitric acid for 48 h. We washed the phalanges in running tap water for 24 h and stained them in Harris’s haematoxylin for 150 min. After that, we dehydrated the stained phalange through successive ethanol stages of 70%, 80%, 95% and 100% for approximately one hour, respectively. We embedded tissues in small paraffin blocks and cut these for phalanges. We then selected cross-sections of about 13 μm thickness with the smallest medullar cavity and mounted them on glass slides. The lines of arrested growths (LAGs) on a section were demonstrated to form in the bones from a cycle of growth and a period of dormancy during hibernation [36]. The number of rings thus relates directly to age, like the rings in trees. We counted LAGs under a light microscope and treated them as the age of the toads because the toads are exposed to distinct temperature cycles through the year. We regarded the first line of arrested growth as endosteal resorption on the basis of the kastschenko line (KL) where there was the interface between the periosteal and endosteal zones [36].

All individuals from the five populations had significant LAGs. Very closely spaced Harris’s haematoxylin lines (double LAGs) were not found for all individuals. False LAGs were observed in one individual, but did not affect the estimate of the age. We did not find endosteal resorption in the sections from males and females. The age of the toad ranged from 1 to 4 in males and 1 to 7 in females, respectively.

### 2.6. Statistical Analysis

We used the R package ‘lme4′ in the RStudio version 4.3.0 [66] to analyze the data. Since there was only one male individual in the Pingbian population, it was not considered in statistical analysis. Before analysis, we performed a log_10_ transformation on the SVL and body mass data to conform to the normality assumption. We first used GLMMs treating age as the dependent variable, altitude or latitude as a fixed factor, sex as a covariate and population as a random factor to analyze geographical variation in age [67]. We then used GLMMs with SVL and body mass as dependent variables, altitude or latitude as a fixed factor, age and sex as covariates, and population as a random factor to test the effect of altitude or latitude on body size variation.

## 3. Results

The number of female and male samples we collected in the five different populations are shown in the table above, which also reflects the mean values of male and female body size among different populations. In populations such as Midu and Pingjiang, where there were a certain number of males and females, the body size of males and females was almost the same. It was noteworthy that the body size of females at the Pingbian population was 72.82 mm, which significantly exceeded the values at other sites.

### 3.1. Geographical Variation in Age

The GLMMs showed that there was a non-significant difference in age among populations and between males and females (Table 2). Meanwhile, the GLMMs showed that the effects of altitude or latitude (Table 2) on age was non-significant (Table 2).

### 3.2. Geographical Variation in Body Size

**Figure 1:**
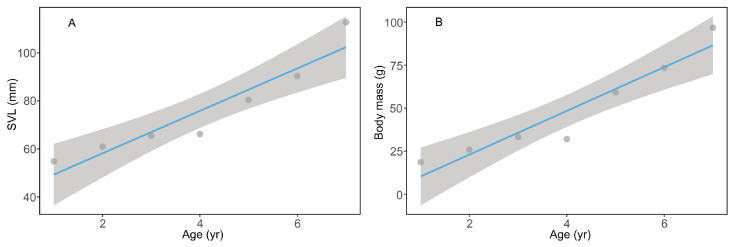
The relationship between age and SVL (**A**) and/or body mass (**B**) among *Duttaphrynus melanostictus* populations.

**Figure 2:**
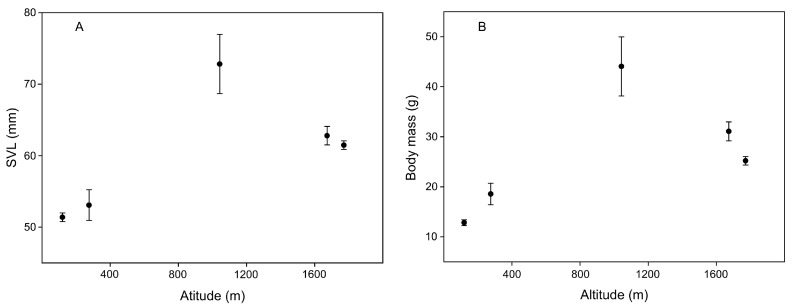
Mean of adult SVL (**A**) and body mass (**B**) changes with increasing altitude across *Duttaphrynus melanostictus* populations. Pingbian population displays the largest body size.

The GLMMs revealed a positive effect of age on body size in *D. melanostictus* across populations (Table 3; Figure 1A,B). Therefore, we need control for age effect to analyze the geographical variation in SVL and body mass across populations. The GLMMs indicated that body size was significantly positively correlated with altitude, and the Pingbian population had the largest body size (Table 3; Figure 2A,B). However, there was a non-significant correlation between body size and latitude (Table 3). The interaction term with altitude and latitude on body size was non-significant (both *p* > 0.05).

## 4. Discussion

Our findings indicate non-significant variation in age across altitudes or latitudes and a positive effect of age on body size. In exploring the effects of different altitudes on the body size of *D. melanostictus*, we found that in populations with a certain number of females and males, the body size of males and females was almost the same. However, there were essentially only females at 1043 m and the body size of *D. melanostictus* at this site was significantly higher than at other higher altitudes. We found that the mean age of *D. melanostictus* at 1043 m was higher than at other higher altitudes and this may result in the body size of *D. melanostictus* at 1043 m being higher compared to at other higher altitudes. It was a clear outlier and we thought there was a positive effect of altitude on body size after controlling for the age effect, following Bergmann’s rule. This pattern suggests that low environmental temperature at high altitude results in larger body size across populations. In the following, we discuss our findings related to previous studies on age and body size variation across populations.

Skeletochronology can be widely used to determine the individual age of amphibians [65,68,69,70,71,72,73,74,75]. We confirmed that skeletochronology can determine the age structure of *D. melanostictus*, although a false line was only found in one individual. In general, the mean age of individuals increases with altitude or latitude among populations in anurans [76,77]. The life-history hypothesis has suggested that high-altitude populations invest more energy in growth by delaying the age at sexual maturity [41]. Indeed, a previous study has found that the mean age of the Andrew’s toad (*Bufo andrewsi*) at high altitudes and/or latitudes is significantly higher than that at low altitudes because the lower environmental temperature at high altitudes leads to later age at sexual maturity and longer lifespan [36]. In this study, there was a non-significant difference in the mean age of *D. melanostictus* at different altitudes or latitudes that was associated with the fact that the difference in length of the breeding season with different altitudes or latitudes was not obvious across populations. This is similar to the results which show that there is a non-significant difference in the mean age of the Andrew’s toad across altitudinal gradients [36].

Geographical variations in body size have been attractive to evolutionary ecologists because it is an important scientific problem in life-history strategy [27,77]. Life-history theory states that age can be an important contributing force to adult body size [38]. Variations in body size along environmental gradients in ectotherms were explored by focusing on Bergmann’s rule [78,79]. Hence, investigating body size variations associated with life-history traits can help understanding the proximate reason for geographic clines in body size. Age and growth rate are two basic life-history traits in animals under natural selection. Cold temperature and limited food availability at high latitudes and/or altitudes are expected to select for slower growth rates, delaying sexual maturity and longer longevity [36]. Consequently, individuals under harsh environments should allocate more time and energy into growth, so that they have larger body size [49]. Herein, although age did not vary consistently with altitude or latitude in the toad, high-altitude populations had larger body size than low-altitude populations. When controlling for age effect, altitudinal variation in body size followed Bergman’s rule, which was driven by population-level adaptation to different thermal environments. This pattern suggested that larger individuals can store more energy to adapt to the cold and highly variable environment so that they live longer and improve their survival rate in adversity. Our findings were consistent with previous studies on geographic variation in body size in some species of frogs conforming to Bergmann’s rule, which show that larger body size is observed in lower ambient temperatures, in addition to longer hibernation periods and shorter activity time at high altitude [36,49]. By contrast, some studies have shown that the body size of anurans species gradually decreases with increasing altitude or latitude [13,50,80].

A previous study has shown that body size of adults is associated with not only age, but also growth rate and size at the starting point of growth in a toad [36]. Indeed, the body size of anurans is known to be positively correlated with age, growth rate and lifespan [80]. There are three parameters (i.e., age, growth rate and lifespan) that mainly determine body size variation shifts along a geographic gradient, yet some anurans support Bergmann’s rule and others obey it. Usually, when selection favors the negative growth rate–longevity correlation for anurans living in contrasting conditions, the relative influences of the two elements associated with local environments determine the cline rule of a species [20,36]. For Bergmann’s rule, later age of maturity and longer lifespan play a more important role for increased body size than slower growth in declining it. On the contrary, any prolonged time taken for growth fails to compensate the influence of slow growth on body size, which will result in the converse of Bergmann’s rule. Indeed, the predominant influence of fast growth rate and long longevity on increased body size along altitudinal gradients exhibits Bergmann’s rule [20,36], whereas the influence of slower growth rate on reduced body size along geographical gradients follows the converse of Bergmann’s rule in *Nanorana parkeri* [53]. Our findings suggested that body size variation in *D. melanostictus* following Bergmann’s rule was likely to be the result of fast growth rates and long longevity in high-altitude populations.

## 5. Conclusions

Consistent with our prediction, our findings offer substantial evidence for the relationship between body size and altitude among populations following Bergmann’s rule. Our findings imply that individuals at high-altitude populations experiencing low temperature have later age at sexual maturity and longer lifespan, which promote the development of larger body size to meet reproductive demands. This pattern suggests that body size variation in *D. melanostictus* can be explained by Bergmann’s rule.

## Figures and Tables

**Table 1:** Sampling size (*n*), altitude, latitude and sex across populations in the Asian common toad (*Duttaphrynus melanostictus*).

Study Site	Latitude (N)	Altitude (m)	Male Body Size (mm)	Female Body Size (mm)
Midu	25°20′	1673	63.06 ± 5.26 (*n* = 33)	62.05 ± 4.34 (*n* = 12)
Mouding	25°17′	1771	61.46 ± 4.33 (*n* = 50)	*n* = 0
Pingjiang	25°58′	276	52.56 ± 4.72 (*n* = 8)	53.59 ± 4.27 (*n* = 8)
Pingbian	22°59′	1043	47.70 (*n* = 1)	72.82 ± 5.08 (*n* = 17)
Yuanling	28°26′	120	51.38 ± 2.55 (*n* = 24)	*n* = 0

**Table 2:** The influences of altitude, latitude and sex on age across populations in the Asian common toad (*Duttaphrynus melanostictus*).

Source	Random			Fixed				
	VAR	SD	Effect Size	Estimate	SE	df	*t*	*p*
Age								
Population	0.492	0.702						
Residual	1.361	1.167						
Altitude			−0.050	<0.001	<0.001	2.275	−0.208	0.853
Sex			−0.170	−0.218	0.306	93.799	−0.712	0.478
Age								
Population	0.294	0.542						
Residual	1.359	1.166						
Latitude			−0.250	−0.219	0.163	3.601	−1.348	0.256
Sex			−0.140	−0.178	0.312	122.224	−0.570	0.570

**Table 3:** The influences of altitude, latitude and sex on body size across populations in the Asian common toad (*Duttaphrynus melanostictus*) after controlling age effect.

Source	Random			Fixed				
	VAR	SD	Effect Size	Estimate	SE	df	*t*	*p*
SVL								
Population	<0.001	0.021						
Residual	0.001	0.030						
Altitude			0.520	<0.001	<0.001	2.744	3.910	0.035
Sex			0.150	0.011	0.008	120.600	1.356	0.178
Age			0.800	0.045	0.002	147.900	21.551	<0.001
Body mass								
Population	0.002	0.045						
Residual	0.011	0.106						
Altitude			0.580	<0.001	<0.001	1.615	5.925	0.044
Sex			0.190	0.042	0.027	48.880	1.553	0.127
Age			0.710	0.124	0.007	146.600	16.754	<0.001
SVL								
Population	0.001	0.037						
Residual	0.001	0.030						
Latitude			−0.350	−0.017	0.010	3.078	−1.760	0.174
Sex			0.200	0.014	0.008	147.639	1.738	0.084
Age			0.800	0.045	0.002	145.855	21.230	<0.001
Body mass								
Population	0.011	0.104						
Residual	0.011	0.106						
Latitude			−0.430	−0.066	0.028	3.131	−2.383	0.094
Sex			0.320	0.072	0.029	147.983	2.448	0.016
Age			0.700	0.121	0.007	146.331	16.124	<0.001

## Data Availability

The data presented in this study are available on request from the corresponding author. The data are not publicly available due to privacy or ethical restrictions.

## Supplementary Materials

The following supporting information can be downloaded at: https://www.mdpi.com/article/10.3390/life13112219/s1, Table S1. The toad museum number information at different collecting sites.
