# Supplementary material for: Geographical Variation in Body Size in the Asian Common Toad (Duttaphrynus melanostictus)
Source: Life (Basel). 2023 Nov 17;13(11):2219. doi: 10.3390/life13112219 (PMC10672612; doi:10.3390/life13112219)
Supplement: Supplementary file 1 [file life-13-02219-s001.zip › life-2576120-supplementary.pdf]

Table S1. The toad museum number information at different collecting sites

Sample site	Longitude (E)	Latitude (N)	Number	Species name	Sex
Midu County, Yunnan	100°29′	25°20′	2018050201	Asian common toad (*Duttaphrynus melanostictus*)	male
Midu County, Yunnan	100°29′	25°20′	2018050202	Asian common toad (*Duttaphrynus melanostictus*)	female
Midu County, Yunnan	100°29′	25°20′	2018050203	Asian common toad (*Duttaphrynus melanostictus*)	male
Midu County, Yunnan	100°29′	25°20′	2018050204	Asian common toad (*Duttaphrynus melanostictus*)	male
Midu County, Yunnan	100°29′	25°20′	2018050205	Asian common toad (*Duttaphrynus melanostictus*)	male
Midu County, Yunnan	100°29′	25°20′	2018050206	Asian common toad (*Duttaphrynus melanostictus*)	female
Midu County, Yunnan	100°29′	25°20′	2018050207	Asian common toad (*Duttaphrynus melanostictus*)	male
Midu County, Yunnan	100°29′	25°20′	2018050208	Asian common toad (*Duttaphrynus melanostictus*)	female
Midu County, Yunnan	100°29′	25°20′	2018050209	Asian common toad (*Duttaphrynus melanostictus*)	male
Midu County, Yunnan	100°29′	25°20′	2018050210	Asian common toad (*Duttaphrynus melanostictus*)	female
Midu County, Yunnan	100°29′	25°20′	2018050211	Asian common toad (*Duttaphrynus melanostictus*)	male
Midu County, Yunnan	100°29′	25°20′	2018050212	Asian common toad (*Duttaphrynus melanostictus*)	male
Midu County, Yunnan	100°29′	25°20′	2018050213	Asian common toad (*Duttaphrynus melanostictus*)	male
Midu County, Yunnan	100°29′	25°20′	2018050214	Asian common toad (*Duttaphrynus melanostictus*)	male
Midu County, Yunnan	100°29′	25°20′	2018050215	Asian common toad (*Duttaphrynus melanostictus*)	male
Midu County, Yunnan	100°29′	25°20′	2018050216	Asian common toad (*Duttaphrynus melanostictus*)	female
Midu County, Yunnan	100°29′	25°20′	2018050217	Asian common toad (*Duttaphrynus melanostictus*)	male
Midu County, Yunnan	100°29′	25°20′	2018050218	Asian common toad (*Duttaphrynus melanostictus*)	female
Midu County, Yunnan	100°29′	25°20′	2018050219	Asian common toad (*Duttaphrynus melanostictus*)	female
Midu County, Yunnan	100°29′	25°20′	2018050220	Asian common toad (*Duttaphrynus melanostictus*)	male
Midu County, Yunnan	100°29′	25°20′	2018050221	Asian common toad (*Duttaphrynus melanostictus*)	male
Midu County, Yunnan	100°29′	25°20′	2018050222	Asian common toad (*Duttaphrynus melanostictus*)	male
Midu County, Yunnan	100°29′	25°20′	2018050223	Asian common toad (*Duttaphrynus melanostictus*)	female
Midu County, Yunnan	100°29′	25°20′	2018050224	Asian common toad (*Duttaphrynus melanostictus*)	male

Midu County, Yunnan	100°29′	25°20′	2018050225	Asian common toad (*Duttaphrynus melanostictus*)	male
Midu County, Yunnan	100°29′	25°20′	2018050226	Asian common toad (*Duttaphrynus melanostictus*)	male
Midu County, Yunnan	100°29′	25°20′	2018050227	Asian common toad (*Duttaphrynus melanostictus*)	male
Midu County, Yunnan	100°29′	25°20′	2018050228	Asian common toad (*Duttaphrynus melanostictus*)	female
Midu County, Yunnan	100°29′	25°20′	2018050229	Asian common toad (*Duttaphrynus melanostictus*)	female
Midu County, Yunnan	100°29′	25°20′	2018050230	Asian common toad (*Duttaphrynus melanostictus*)	male
Midu County, Yunnan	100°29′	25°20′	2018050231	Asian common toad (*Duttaphrynus melanostictus*)	male
Midu County, Yunnan	100°29′	25°20′	2018050232	Asian common toad (*Duttaphrynus melanostictus*)	male
Midu County, Yunnan	100°29′	25°20′	2018050233	Asian common toad (*Duttaphrynus melanostictus*)	female
Midu County, Yunnan	100°29′	25°20′	2018050234	Asian common toad (*Duttaphrynus melanostictus*)	male
Midu County, Yunnan	100°29′	25°20′	2018050235	Asian common toad (*Duttaphrynus melanostictus*)	male
Midu County, Yunnan	100°29′	25°20′	2018050236	Asian common toad (*Duttaphrynus melanostictus*)	male
Midu County, Yunnan	100°29′	25°20′	2018050237	Asian common toad (*Duttaphrynus melanostictus*)	male
Midu County, Yunnan	100°29′	25°20′	2018050238	Asian common toad (*Duttaphrynus melanostictus*)	female
Midu County, Yunnan	100°29′	25°20′	2018050239	Asian common toad (*Duttaphrynus melanostictus*)	male
Midu County, Yunnan	100°29′	25°20′	2018050240	Asian common toad (*Duttaphrynus melanostictus*)	male
Midu County, Yunnan	100°29′	25°20′	2018050241	Asian common toad (*Duttaphrynus melanostictus*)	male
Midu County, Yunnan	100°29′	25°20′	2018050242	Asian common toad (*Duttaphrynus melanostictus*)	male
Midu County, Yunnan	100°29′	25°20′	2018050243	Asian common toad (*Duttaphrynus melanostictus*)	male
Midu County, Yunnan	100°29′	25°20′	2018050244	Asian common toad (*Duttaphrynus melanostictus*)	male
Midu County, Yunnan	100°29′	25°20′	2018050245	Asian common toad (*Duttaphrynus melanostictus*)	male
Mouding County, Yunnan	101°33′	25°17′	2019040501	Asian common toad (*Duttaphrynus melanostictus*)	male
Mouding County, Yunnan	101°33′	25°17′	2019040502	Asian common toad (*Duttaphrynus melanostictus*)	male
Mouding County, Yunnan	101°33′	25°17′	2019040503	Asian common toad (*Duttaphrynus melanostictus*)	male
Mouding County, Yunnan	101°33′	25°17′	2019040504	Asian common toad (*Duttaphrynus melanostictus*)	male
Mouding County, Yunnan	101°33′	25°17′	2019040505	Asian common toad (*Duttaphrynus melanostictus*)	male

Mouding County, Yunnan	101°33′	25°17′	2019040506	Asian common toad (*Duttaphrynus melanostictus*)	male
Mouding County, Yunnan	101°33′	25°17′	2019040507	Asian common toad (*Duttaphrynus melanostictus*)	male
Mouding County, Yunnan	101°33′	25°17′	2019040508	Asian common toad (*Duttaphrynus melanostictus*)	male
Mouding County, Yunnan	101°33′	25°17′	2019040509	Asian common toad (*Duttaphrynus melanostictus*)	male
Mouding County, Yunnan	101°33′	25°17′	2019040510	Asian common toad (*Duttaphrynus melanostictus*)	male
Mouding County, Yunnan	101°33′	25°17′	2019040511	Asian common toad (*Duttaphrynus melanostictus*)	male
Mouding County, Yunnan	101°33′	25°17′	2019040512	Asian common toad (*Duttaphrynus melanostictus*)	male
Mouding County, Yunnan	101°33′	25°17′	2019040513	Asian common toad (*Duttaphrynus melanostictus*)	male
Mouding County, Yunnan	101°33′	25°17′	2019040514	Asian common toad (*Duttaphrynus melanostictus*)	male
Mouding County, Yunnan	101°33′	25°17′	2019040515	Asian common toad (*Duttaphrynus melanostictus*)	male
Mouding County, Yunnan	101°33′	25°17′	2019040516	Asian common toad (*Duttaphrynus melanostictus*)	male
Mouding County, Yunnan	101°33′	25°17′	2019040517	Asian common toad (*Duttaphrynus melanostictus*)	male
Mouding County, Yunnan	101°33′	25°17′	2019040518	Asian common toad (*Duttaphrynus melanostictus*)	male
Mouding County, Yunnan	101°33′	25°17′	2019040519	Asian common toad (*Duttaphrynus melanostictus*)	male
Mouding County, Yunnan	101°33′	25°17′	2019040520	Asian common toad (*Duttaphrynus melanostictus*)	male
Mouding County, Yunnan	101°33′	25°17′	2019040521	Asian common toad (*Duttaphrynus melanostictus*)	male
Mouding County, Yunnan	101°33′	25°17′	2019040522	Asian common toad (*Duttaphrynus melanostictus*)	male
Mouding County, Yunnan	101°33′	25°17′	2019040523	Asian common toad (*Duttaphrynus melanostictus*)	male
Mouding County, Yunnan	101°33′	25°17′	2019040524	Asian common toad (*Duttaphrynus melanostictus*)	male
Mouding County, Yunnan	101°33′	25°17′	2019040525	Asian common toad (*Duttaphrynus melanostictus*)	male
Mouding County, Yunnan	101°33′	25°17′	2019040526	Asian common toad (*Duttaphrynus melanostictus*)	male
Mouding County, Yunnan	101°33′	25°17′	2019040527	Asian common toad (*Duttaphrynus melanostictus*)	male
Mouding County, Yunnan	101°33′	25°17′	2019040528	Asian common toad (*Duttaphrynus melanostictus*)	male
Mouding County, Yunnan	101°33′	25°17′	2019040529	Asian common toad (*Duttaphrynus melanostictus*)	male
Mouding County, Yunnan	101°33′	25°17′	2019040530	Asian common toad (*Duttaphrynus melanostictus*)	male
Mouding County, Yunnan	101°33′	25°17′	2019040531	Asian common toad (*Duttaphrynus melanostictus*)	male

Mouding County, Yunnan	101°33′	25°17′	2019040532	Asian common toad (*Duttaphrynus melanostictus*)	male
Mouding County, Yunnan	101°33′	25°17′	2019040533	Asian common toad (*Duttaphrynus melanostictus*)	male
Mouding County, Yunnan	101°33′	25°17′	2019040534	Asian common toad (*Duttaphrynus melanostictus*)	male
Mouding County, Yunnan	101°33′	25°17′	2019040535	Asian common toad (*Duttaphrynus melanostictus*)	male
Mouding County, Yunnan	101°33′	25°17′	2019040536	Asian common toad (*Duttaphrynus melanostictus*)	male
Mouding County, Yunnan	101°33′	25°17′	2019040537	Asian common toad (*Duttaphrynus melanostictus*)	male
Mouding County, Yunnan	101°33′	25°17′	2019040538	Asian common toad (*Duttaphrynus melanostictus*)	male
Mouding County, Yunnan	101°33′	25°17′	2019040539	Asian common toad (*Duttaphrynus melanostictus*)	male
Mouding County, Yunnan	101°33′	25°17′	2019040540	Asian common toad (*Duttaphrynus melanostictus*)	male
Mouding County, Yunnan	101°33′	25°17′	2019040541	Asian common toad (*Duttaphrynus melanostictus*)	male
Mouding County, Yunnan	101°33′	25°17′	2019040542	Asian common toad (*Duttaphrynus melanostictus*)	male
Mouding County, Yunnan	101°33′	25°17′	2019040543	Asian common toad (*Duttaphrynus melanostictus*)	male
Mouding County, Yunnan	101°33′	25°17′	2019040544	Asian common toad (*Duttaphrynus melanostictus*)	male
Mouding County, Yunnan	101°33′	25°17′	2019040545	Asian common toad (*Duttaphrynus melanostictus*)	male
Mouding County, Yunnan	101°33′	25°17′	2019040546	Asian common toad (*Duttaphrynus melanostictus*)	male
Mouding County, Yunnan	101°33′	25°17′	2019040547	Asian common toad (*Duttaphrynus melanostictus*)	male
Mouding County, Yunnan	101°33′	25°17′	2019040548	Asian common toad (*Duttaphrynus melanostictus*)	male
Mouding County, Yunnan	101°33′	25°17′	2019040549	Asian common toad (*Duttaphrynus melanostictus*)	male
Mouding County, Yunnan	101°33′	25°17′	2019040550	Asian common toad (*Duttaphrynus melanostictus*)	male
Pingjiang County, Guizhou	108°24′	25°58′	2020042501	Asian common toad (*Duttaphrynus melanostictus*)	male
Pingjiang County, Guizhou	108°24′	25°58′	2020042502	Asian common toad (*Duttaphrynus melanostictus*)	female
Pingjiang County, Guizhou	108°24′	25°58′	2020042503	Asian common toad (*Duttaphrynus melanostictus*)	female
Pingjiang County, Guizhou	108°24′	25°58′	2020042504	Asian common toad (*Duttaphrynus melanostictus*)	male
Pingjiang County, Guizhou	108°24′	25°58′	2020042505	Asian common toad (*Duttaphrynus melanostictus*)	male
Pingjiang County, Guizhou	108°24′	25°58′	2020042506	Asian common toad (*Duttaphrynus melanostictus*)	female
Pingjiang County, Guizhou	108°24′	25°58′	2020042507	Asian common toad (*Duttaphrynus melanostictus*)	female

Pingjiang County, Guizhou	108°24′	25°58′	2020042508	Asian common toad (*Duttaphrynus melanostictus*)	male
Pingjiang County, Guizhou	108°24′	25°58′	2020042509	Asian common toad (*Duttaphrynus melanostictus*)	male
Pingjiang County, Guizhou	108°24′	25°58′	2020042510	Asian common toad (*Duttaphrynus melanostictus*)	male
Pingjiang County, Guizhou	108°24′	25°58′	2020042511	Asian common toad (*Duttaphrynus melanostictus*)	female
Pingjiang County, Guizhou	108°24′	25°58′	2020042512	Asian common toad (*Duttaphrynus melanostictus*)	female
Pingjiang County, Guizhou	108°24′	25°58′	2020042513	Asian common toad (*Duttaphrynus melanostictus*)	female
Pingjiang County, Guizhou	108°24′	25°58′	2020042514	Asian common toad (*Duttaphrynus melanostictus*)	female
Pingjiang County, Guizhou	108°24′	25°58′	2020042515	Asian common toad (*Duttaphrynus melanostictus*)	male
Pingjiang County, Guizhou	108°24′	25°58′	2020042516	Asian common toad (*Duttaphrynus melanostictus*)	male
Pingbian County, Yunnan	103°42′	22°59′	2020061501	Asian common toad (*Duttaphrynus melanostictus*)	female
Pingbian County, Yunnan	103°42′	22°59′	2020061502	Asian common toad (*Duttaphrynus melanostictus*)	female
Pingbian County, Yunnan	103°42′	22°59′	2020061503	Asian common toad (*Duttaphrynus melanostictus*)	female
Pingbian County, Yunnan	103°42′	22°59′	2020061504	Asian common toad (*Duttaphrynus melanostictus*)	female
Pingbian County, Yunnan	103°42′	22°59′	2020061505	Asian common toad (*Duttaphrynus melanostictus*)	female
Pingbian County, Yunnan	103°42′	22°59′	2020061506	Asian common toad (*Duttaphrynus melanostictus*)	male
Pingbian County, Yunnan	103°42′	22°59′	2020061507	Asian common toad (*Duttaphrynus melanostictus*)	female
Pingbian County, Yunnan	103°42′	22°59′	2020061508	Asian common toad (*Duttaphrynus melanostictus*)	female
Pingbian County, Yunnan	103°42′	22°59′	2020061509	Asian common toad (*Duttaphrynus melanostictus*)	female
Pingbian County, Yunnan	103°42′	22°59′	2020061510	Asian common toad (*Duttaphrynus melanostictus*)	female
Pingbian County, Yunnan	103°42′	22°59′	2020061511	Asian common toad (*Duttaphrynus melanostictus*)	female
Pingbian County, Yunnan	103°42′	22°59′	2020061512	Asian common toad (*Duttaphrynus melanostictus*)	female
Pingbian County, Yunnan	103°42′	22°59′	2020061513	Asian common toad (*Duttaphrynus melanostictus*)	female
Pingbian County, Yunnan	103°42′	22°59′	2020061514	Asian common toad (*Duttaphrynus melanostictus*)	female
Pingbian County, Yunnan	103°42′	22°59′	2020061515	Asian common toad (*Duttaphrynus melanostictus*)	female
Pingbian County, Yunnan	103°42′	22°59′	2020061516	Asian common toad (*Duttaphrynus melanostictus*)	female
Pingbian County, Yunnan	103°42′	22°59′	2020061517	Asian common toad (*Duttaphrynus melanostictus*)	female

Pingbian County, Yunnan	103°42′	22°59′	2020061518	Asian common toad (*Duttaphrynus melanostictus*)	female
Yuanling County, Hunan	110°25′	28°26′	2020070801	Asian common toad (*Duttaphrynus melanostictus*)	male
Yuanling County, Hunan	110°25′	28°26′	2020070802	Asian common toad (*Duttaphrynus melanostictus*)	male
Yuanling County, Hunan	110°25′	28°26′	2020070803	Asian common toad (*Duttaphrynus melanostictus*)	male
Yuanling County, Hunan	110°25′	28°26′	2020070804	Asian common toad (*Duttaphrynus melanostictus*)	male
Yuanling County, Hunan	110°25′	28°26′	2020070805	Asian common toad (*Duttaphrynus melanostictus*)	male
Yuanling County, Hunan	110°25′	28°26′	2020070806	Asian common toad (*Duttaphrynus melanostictus*)	male
Yuanling County, Hunan	110°25′	28°26′	2020070807	Asian common toad (*Duttaphrynus melanostictus*)	male
Yuanling County, Hunan	110°25′	28°26′	2020070808	Asian common toad (*Duttaphrynus melanostictus*)	male
Yuanling County, Hunan	110°25′	28°26′	2020070809	Asian common toad (*Duttaphrynus melanostictus*)	male
Yuanling County, Hunan	110°25′	28°26′	2020070810	Asian common toad (*Duttaphrynus melanostictus*)	male
Yuanling County, Hunan	110°25′	28°26′	2020070811	Asian common toad (*Duttaphrynus melanostictus*)	male
Yuanling County, Hunan	110°25′	28°26′	2020070812	Asian common toad (*Duttaphrynus melanostictus*)	male
Yuanling County, Hunan	110°25′	28°26′	2020070813	Asian common toad (*Duttaphrynus melanostictus*)	male
Yuanling County, Hunan	110°25′	28°26′	2020070814	Asian common toad (*Duttaphrynus melanostictus*)	male
Yuanling County, Hunan	110°25′	28°26′	2020070815	Asian common toad (*Duttaphrynus melanostictus*)	male
Yuanling County, Hunan	110°25′	28°26′	2020070816	Asian common toad (*Duttaphrynus melanostictus*)	male
Yuanling County, Hunan	110°25′	28°26′	2020070817	Asian common toad (*Duttaphrynus melanostictus*)	male
Yuanling County, Hunan	110°25′	28°26′	2020070818	Asian common toad (*Duttaphrynus melanostictus*)	male
Yuanling County, Hunan	110°25′	28°26′	2020070819	Asian common toad (*Duttaphrynus melanostictus*)	male
Yuanling County, Hunan	110°25′	28°26′	2020070820	Asian common toad (*Duttaphrynus melanostictus*)	male
Yuanling County, Hunan	110°25′	28°26′	2020070821	Asian common toad (*Duttaphrynus melanostictus*)	male
Yuanling County, Hunan	110°25′	28°26′	2020070822	Asian common toad (*Duttaphrynus melanostictus*)	male
Yuanling County, Hunan	110°25′	28°26′	2020070823	Asian common toad (*Duttaphrynus melanostictus*)	male
Yuanling County, Hunan	110°25′	28°26′	2020070824	Asian common toad (*Duttaphrynus melanostictus*)	male
